# Data on medicinal plants used in Central America to manage diabetes and its sequelae (skin conditions, cardiovascular disease, kidney disease, urinary problems and vision loss)

**DOI:** 10.1016/j.dib.2016.03.102

**Published:** 2016-04-06

**Authors:** Peter Giovannini, Melanie-Jayne R. Howes, Sarah E. Edwards

**Affiliations:** a Natural Capital and Plant Health Department, Royal Botanic Gardens Kew, Wakehurst Place, Ardingly, West Sussex RH17 6TN, UK; b Centre for Biocultural Diversity, School of Anthropology and Conservation, Marlowe Building, University of Kent, Canterbury, Kent CT2 7NR, UK; c Natural Capital and Plant Health Department, Jodrell Laboratory, Royal Botanic Gardens Kew, Richmond, Surrey TW9 3AB, UK; d Institute of Pharmaceutical Science, Faculty of Life Sciences & Medicine, Franklin-Wilkins Building, King’s College London, 150 Stamford Street, London SE1 9NH, UK; e Medicinal Plant Name Services, Royal Botanic Gardens Kew, Richmond, Surrey TW9 3AB, UK; f Center for Pharmacognosy & Phytotherapy, UCL School of Pharmacy, Univ. London, 29-39 Brunswick Sq., London WC1N 1AX, UK

**Keywords:** Diabetes, Central America, Medicinal plants, Hypoglycemic, Traditional medicine, Herbal remedies, Ecosystem services, Natural capital

## Abstract

The data described in this article is related to the review article “Medicinal plants used in the traditional management of diabetes and its sequelae in Central America: a review” (Giovannini et al., 2016) [1]. We searched publications on the useful plants of Central America in databases and journals by using selected relevant keywords. We then extracted reported uses of medicinal plants within the disease categories: diabetes mellitus, kidney disease, urinary problems, skin diseases and infections, cardiovascular disease, sexual dysfunction, vision loss, and nerve damage. The following countries were included in our definition of Central America: Belize, Guatemala, Honduras, El Salvador, Nicaragua, Costa Rica and Panama. Data were compiled in a bespoke Access database. Plant names from the published sources were validated against The Plant List (TPL, (The Plant List, 2013) [2]) and accepted names and synonyms were extracted. In total, the database includes 607 plant names obtained from the published sources which correspond to 537 plant taxa, 9271 synonyms and 1055 use reports.

**Specifications Table**

**Table:** 

Subject area	Biology, Pharmacology
More specific subject area	Ethnobotany and Ethnopharmacology
Type of data	Table, figure, Access database
How data was acquired	Literature review of published primary data
Data format	Coded, filtered, and analyzed
Experimental factors	Primary data on the useful plants of Central America
Experimental features	Publication with primary data searched using both English and Spanish Keywords in databases. Keywords: name of the country and “ethnobotany”, “medicinal plants”, “ethnopharmacology”, “ethnomedicine”, and “herbal medicine”.
Data source location	Royal Botanic Gardens Kew, UK
Data accessibility	Data is within this article

**Value of the data**

• Data scattered across literature compiled in one database.
• Future research and analysis on the medicinal plants used to manage diabetes and its sequelae at country and regional level will be facilitated by the data included here.
• Plant names validated against The Plant List, taxonomic status checked, and current accepted name provided.
• Complete list of synonyms for compiled medicinal plants to facilitate research.

## 1 Data

The data includes 1055 use reports of 537 plant taxa used to manage diabetes and its sequelae in Central America (Supplementary material 1: table MedicinalPlants_ReferencesUseReports). These use reports were extracted from 32 sources publishing primary data on the useful plants of Central America (Supplementary material 1: table References). The data also include the plant names as originally entered in the database, the name of these were matched to The Plant List (TPL), and the accepted name according to TPL (Supplementary material 1: table Medicinal Plants_matched_TPL); and a full list of synonyms (9271 records) for each accepted name (Supplementary material 1: table MedicinalPlants_Synonyms). TPL identifiers, taxonomic status, data source, International Plant Name Index (IPNI) identifiers and confidence levels (see [2]) are also provided in the tables. The data also include tables of analysis of numbers of use reports by family, genus and full accepted name (Supplementary material 1).

The data are analysed in a related article [1].

## 2 Experimental design, materials and methods

**Fig. 1:**
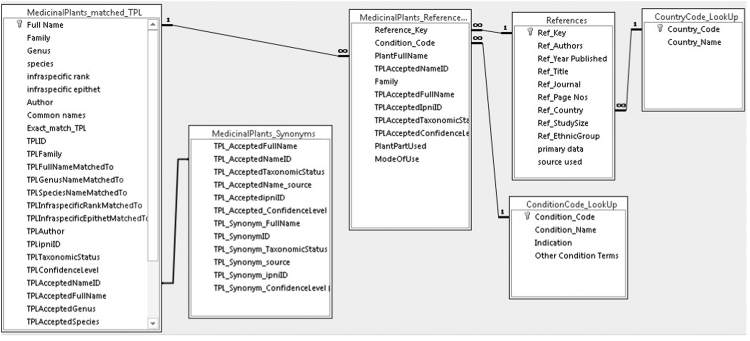
Diagram showing the relationships among the tables within the database.

We searched publications with data on the useful plants of Central America in databases (SCOPUS, Web of Science, Google Scholar, and PubMed) and on relevant journals by using selected relevant keywords (name of the country and “ethnobotany”, “medicinal plants”, “ethnopharmacology”, “ethnomedicine”, and “herbal medicine”). We conducted the search using both English and Spanish Keywords. Then, we extracted reported uses of medicinal plants and entered the data in a bespoke Access database within the disease categories diabetes mellitus, kidney disease, urinary problems, skin diseases and infections, cardiovascular disease, sexual dysfunctions, visual loss, and nerve damage. We included in our definition of Central America the following countries: Belize, Guatemala, Honduras, El Salvador, Nicaragua, Costa Rica and Panama. Compiled data were entered in a bespoke Access database (Supplementary material 1). Table 1 shows the structure of the database and Fig. 1 shows the relationships among the tables within the database. Only primary data was extracted from literature and included in the database. Plant names from the published sources were validated against The Plant List (TPL, [2]) at point of entry and, after data entry, by evaluating automatically the entire dataset against TPL. Where synonyms were found in the primary sources these were matched to the accepted name according to TPL, to avoid miscounting the number of plant taxa found, as some plants were found under more than one name. Complete lists of synonyms for each accepted plant name were extracted from TPL.

## Figures and Tables

**Table 1:** Structure of the database of medicinal plants used in the traditional management of diabetes and its sequelae in Central America.

**Name of the table**	**Content**	**Fields in the table**
Analysis_by_Accepted_Genus	Results of the analysis of use reports at genus level	Family; TPL accepted genus; total # of uses; reports for each disease category (CD, DM, KD, SI, UP, VL)
Analysis_by_family_no_species	Results of the analysis of use reports at family level	Family; Total species in TPL; species in database; total use reports; reports for each disease category (CD, DM, KD, SI, UP, VL)
Analysis_UseReports_by_Accepted_FullName	Results of the analysis of use reports at species level	Species accepted full name; total # of use reports; reports for each disease category (CD, DM, KD, SI, UP, VL)
ConditionCode_Lookup	Codes for disease category used in the database	CD (cardiovascular disease); DM (Diabetes mellitus); KD (Kidney disease); ND (Nerve Damage); SD (Sexual dysfunction); SI (Skin infection/disease); UP (Urinary problems); VL (Vision loss)
CountryCode_LookUP	Codes for countries used in the database	BE (Belize); CA (Central America); CR (Costa Rica); ES (EL Salvador); GU (Guatemala); HO (Honduras); NI (Nicaragua); Pa (Panama)
MedicinalPlants_matched_TPL	Plant names collated from references matched to The Plant List (TPL)	Medicinal plant full name; family; genus; species; infraspecific rank; infraspecific epithet; plant name author; exact match to TPL (Y/N); TPL record ID; TPL family; TPL full name matched to; TPL genus matched to; TPL species matched to; TPL plant name authors; International Plant Name Index (ipni) ID; Taxonomic status of name according to TPL v1.1; Confidence level of name: H = High (3-star, globally peer-reviewed), M= Medium, L= Low; TPL accepted name identifier; TPL accepted full name; TPL accepted genus; TPL accepted species; TPL accepted infraspecific rank; TPL accepted infraspecific epithet; TPL accepted name authors; Accepted name ipni ID; Taxonomic status of linked ׳accepted׳ name; Confidence level of ׳accepted׳ name
MedicinalPlants_ReferenceUseReports	Use reports extracted from literature	Reference key; Condition; plant full name (prior to name validation); TPL accepted name ID; Family; TPL accepted full name; accepted name ipni ID; TPL ‘accepted’ name taxonomic status; TPL accepted name confidence level; Plant part used; Mode of use
MedicinaPlants_Synonyms	Complete list of synonyms found in TPL for each medicinal plant in dataset	TPL accepted name; TPL accepted name ID; TPL accepted name taxonomic status; TPL name data source; accepted name ipni ID; TPL accepted name confidence level; TPL synonym full name; TPL synonym ID; TPL synonym taxonomic status; TPL synonym name source; TPL synonym ipni ID; TPL synonym confidence level
References	Sources consulted or from which data was extracted	Reference key; Reference authors; Year of publication ; Title; Journal; volume, issue and page numbers; country; study size; ethnic group studied; primary data; source used
